# Osseous metaplasia in an adenocarcinoma of the cecum: A rare case report

**DOI:** 10.1016/j.ijscr.2025.112021

**Published:** 2025-10-06

**Authors:** Seblewengel Maru Wubalem, Birhanu Kassie Reta, Woldie Jember Zewdie, Shemsu Abraham Hussien

**Affiliations:** a Department of Pathology, Wachemo University, Hossana, Ethiopia; b Department of Pathology, Aksum University, Aksum, Ethiopia; c Department of Pathology, Worabe Comprehensive Specialized Hospital, Worabe, Ethiopia

**Keywords:** Cecum, Colon, Adenocarcinoma, Heterotopic ossification, Osseous metaplasia

## Abstract

**Introduction:**

Osseous metaplasia (OM) is a rare condition characterized by formation of bone outside of the skeletal system. It is a rare phenomenon in the gastrointestinal tract. OM in colon cancer was first reported by Duckes in 1939. The aim of this case report is to highlight the possible occurrence of OM in colorectal carcinoma.

**Case presentation:**

A 46-year-old female patient known to have type two diabetes mellitus presented to the emergency department with a complaint of abdominal pain lasting 15 days. Her abdomen-pelvic ultrasound revealed a right lower quadrant focal collection. Right hemicolectomy with abscess drainage was performed with an assessment of appendiceal abscess. Histologic examination showed well-differentiated adenocarcinoma with osseous metaplasia.

**Discussion:**

OM has been observed in the gastrointestinal tract, linked to benign gastric and colonic polyps, adenomas, and various carcinomas, especially in the rectum. It typically affects individuals aged 32 to 90 and is frequently associated with well to moderately differentiated adenocarcinomas. The mechanisms behind metaplastic bone formation in these lesions are unclear, though overexpression of osteogenic factors like bone morphogenetic proteins (BMPs) is suspected. OM can mimic mesenchymal tumors, necessitating careful histopathological examination to distinguish it from metastasis.

**Conclusion:**

To our knowledge, this is the first report of OM in colon adenocarcinoma in an Ethiopian patient. It should be differentiated from osteosarcoma and metastasis of carcinoma to the bone, as the latter two have worse prognoses. Further studies are necessary to determine its etiology, pathogenesis, and prognostic significance of OM in colon adenocarcinoma.

## Highlights

• Osseous metaplasia (OM) is a rare condition in the gastrointestinal tract.
• Presence of OM in the colon cancer raises the possibility of osteosarcoma or metastasis to the bone.
• More studies are needed to determine the pathogenesis and prognosis of OM in colon adenocarcinoma.

## 1 Introduction

Osseous metaplasia (OM), also known as heterotopic ossification, is a rare condition characterized by the formation of bone outside of the skeletal system. OM is an uncommon phenomenon in the gastrointestinal tract (GIT) and can occur in any part of the GIT, from the esophagus to the rectum [1], including the gallbladder [2]. It is associated with both non-neoplastic [3,4] and neoplastic conditions [[5], [6], [7]]. It was first reported by Duckes in 1939. Duckes found the incidence of OM in colorectal malignancy to be less than 0.4 % [8]. We present a case of OM in colonic adenocarcinoma in a 46-year-old female patient. The aim of this case report is to highlight the possible occurrence of OM in colorectal carcinoma. This case report has been reported in line with the SCARE checklist [22].

## 2 Case presentation

This case involves a 46-year-old female patient with a three-year history of type 2 diabetes mellitus, well-controlled with metformin, glibenclamide, and atorvastatin. She underwent cholecystectomy five years ago due to chronic cholecystitis secondary to cholelithiasis. On August 5, 2023, she presented to the emergency department with a complaint of abdominal pain that had lasted for 15 days. The pain, initially mild, progressively intensified. She also reported associated symptoms of nausea, vomiting, loss of appetite, low-grade intermittent fever, malaise, chills, and rigors. Upon examination, she appeared acutely ill, exhibiting tachycardia and borderline blood pressure (pulse rate: 130 bpm, blood pressure: 94/59 mmHg, respiratory rate: 18 breaths/min, oxygen saturation: 97 %). Abdominal examination revealed tenderness in the right lower quadrant (Table 1).

**Table 1:** Time line of the clinical course. (T = time).

Time of hospital visit	Clinical findings and management
T0 (initial visit)	With an assessment of acute abdomen secondary to appendiceal abscess, septic shock, prerenal azotemia, and mild anemia in a patient with known type 2 diabetes mellitus, she was started on ceftriaxone and metronidazole, resuscitated with three bags of fluid, and prepared for surgery.
	Intraoperatively, after draining the abscess through Rocky-Davis incision, cecal perforation was visualized. Therefore, a midline vertical incision was made, the peritoneum was entered, and the cecum and ascending colon were immobilized. A right hemicolectomy with ileo-transverse anastomosis was performed.
T1 (immediate postoperative day)	She was transferred to the intensive care unit. The medications were revised to ceftazidime, metronidazole, and vancomycin. She was placed on maintenance fluid and a sliding scale for insulin. Additionally, she was transfused with two packs of blood.
T2 (second postoperative day)	The septic shock was corrected, and the WBC count dropped to normal. The renal function tests also showed improvement. Therefore, she was transferred to the surgical ward.
T3 (third postoperative day)	The repeated renal function test showed further improvement. However, she developed mild hypokalemia (potassium = 2.8 mmol/L), and intravenous KCl was initiated.
T4 (fourth postoperative day)	The patient passed away on the fourth postoperative day due to sudden cardiorespiratory arrest; the possible cause was speculated to be pulmonary embolism.

Investigations showed leukocytosis in the complete blood count (WBC = 14.9 × 10^3^/μL) and mild anemia (hemoglobin = 11.5 g/dL). Renal function tests indicated elevated creatinine and urea levels (creatinine = 1.9 mg/dL, urea = 78.11 mg/dL), while liver function tests were within normal limits. An abdomen-pelvic ultrasound revealed a focal collection in the right lower quadrant measuring 10 cm × 9 cm, along with mesenteric fat stranding at that site. The appendix was not visualized, and other bowel segments appeared normal in caliber and wall thickness, with no mass lesions detected. The ultrasound findings suggested an appendiceal abscess.

Given the assessment of acute abdomen secondary to an appendiceal abscess, septic shock, prerenal azotemia, and mild anemia in a patient with known type 2 diabetes mellitus, she was initiated on ceftriaxone and metronidazole, resuscitated with three bags of fluids, and prepared for surgery.

Intraoperatively, a Rocky-Davis incision was made to enter the abdomen through the right lower quadrant (RLQ). Omental migration and a pocket of abscess cavity were found between the posterolateral abdominal wall and the cecum, from which 100 mL of thick, foul-smelling pus was aspirated. The appendix was necrotic, and cecal perforation was observed. A midline vertical incision was then made, and the peritoneum was entered, allowing for immobilization of the cecum and ascending colon. A right hemicolectomy with ileo-transverse anastomosis was performed. The peritoneum was lavaged, a drain was placed in the RLQ, and the abdomen was closed in layers.

Following the procedure, she was transferred to the intensive care unit. Her medications were adjusted to ceftazidime, metronidazole, and vancomycin. She was placed on maintenance fluids and a sliding scale for insulin, and two units of blood were transfused. By the second postoperative day, her septic shock had resolved (BP = 123/84 mmHg), and her WBC count had decreased to 10.1 × 10^3^/μL. Renal function tests also showed improvement (creatinine = 1.81 mg/dL, urea = 73.09 mg/dL), leading to her transfer to the surgical ward.

In the surgical ward, the previous management continued. On the third postoperative day, renal function tests showed further improvement (creatinine = 1.73 mg/dL, urea = 69.08 mg/dL). However, she developed mild hypokalemia (potassium = 2.8 mmol/L), prompting the initiation of intravenous KCl. The patient passed away on the fourth postoperative day due to sudden cardiorespiratory arrest, with pulmonary embolism speculated as a possible cause. A metastatic workup was not performed since the CT scan was non-functional at the time, and priority was given to stabilizing her condition and controlling the infection before further investigations.

**Fig. 1:**
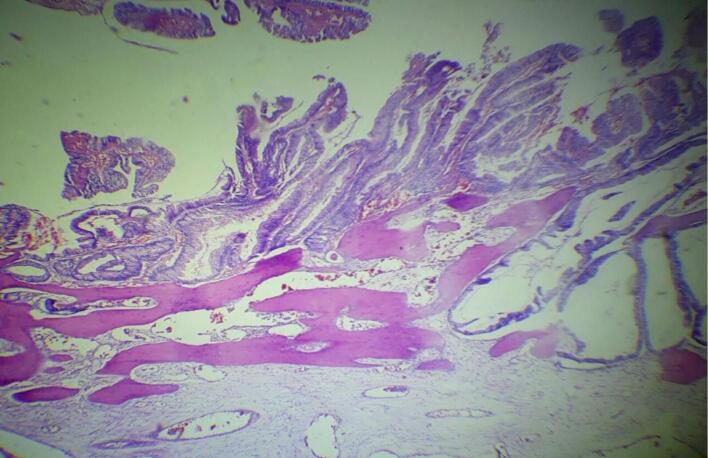
Histologic sections show surface villous adenoma with high grade dysplasia. Underneath there are invasive malignant glands and heterotopic bone formation (Hematoxilin and Eosin, 4×)

**Fig. 2:**
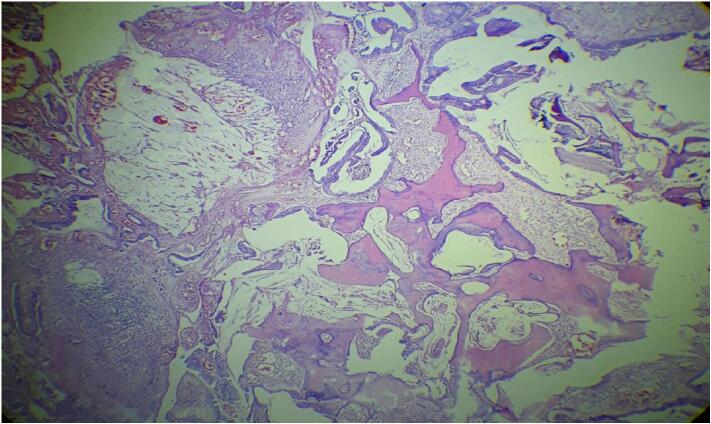
Histologic sections reveal invasive malignant glands with osseous metaplasia. Mucin pool (upper left) and necrosis (lower left) seen. (Hematoxilin and Eosin, 10×).

**Fig. 3:**
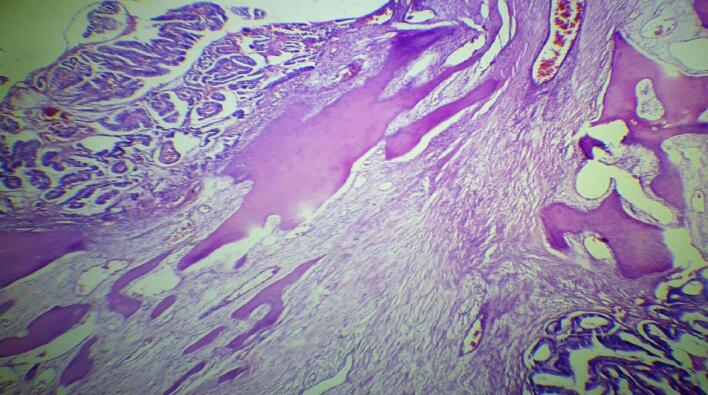
Histologic sections show osseous metaplasia with surrounding fibrosed stroma. (Hematoxilin and Eosin, 10×).

**Fig. 4:**
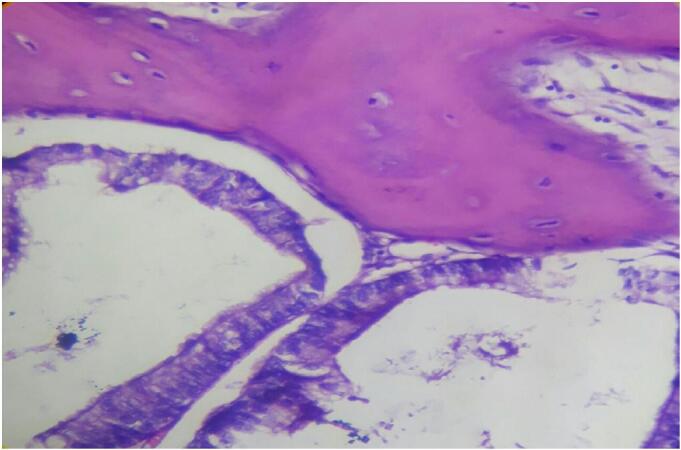
Histologic sections demonstrate heterotopic bone containing bland osteocytes in the lacunar spaces. (Hematoxilin and Eosin, 40×).

Gross examination the resected specimen revealed a 33 cm long segment of bowel and a 10 × 2 cm attached mesentery with a 4 × 4 cm cecal perforation. No lymph nodes were identified. Histopathological examination of Hematoxylin and Eosin-stained sections showed colonic mucosa lining with a villous adenoma exhibiting high-grade dysplasia. Beneath this, nests of malignant glands were invading the submucosa and muscularis propria (Fig. 1). Areas of fibrosis, necrosis, mucin pools, and chronic inflammatory infiltrates were also noted (Fig. 2, Fig. 3). The mucin constituted less than 50 % of the tumor. Heterotopic bone formation surrounded by fibrous stroma and malignant glands was observed, with the heterotopic bone containing bland osteocytes in lacunar spaces without osteoblastic rimming or marrow elements (Fig. 4). No lymphovascular or perineural invasion was noted, and proximal and distal resection margins were free of tumor. Given these findings, the diagnosis was made of well-differentiated adenocarcinoma with osseous metaplasia. The status of the mesenteric lymph nodes and metastasis was not assessed (PT4a N × M×).

## 3 Discussion

OM has been reported in the GIT in association with benign gastric and colonic polyps, adenomas and carcinomas [1,5,6,[9], [10], [11], [12], [13]]. It has also been observed in metastatic colonic carcinoma to the lymph nodes [14,15]. The rectum is the most common site of OM that is associated with carcinoma [7]. The age range of OM with colonic carcinoma is 32 to 90 years old [5,14]. It is frequently seen in well to moderately differentiated adenocarcinomas [5,7].

The etiology and pathogenesis of metaplastic bone formation are not yet well understood. The common associated histopathologic findings include mucin production, [4] necrosis [8] and desmoplastic stroma [16,17]. In our case, we observed all three features. Additionally, we noted inflammatory infiltrate; however, existing studies do not demonstrate their association with OM in colonic carcinoma, highlighting the need for further research.

The formation of bone in GIT lesions has been speculated to be due to the overexpression of osteogenic factors like bone morphogenetic protein (BMP) and osteocalcin [18]. BMP belongs to the transforming growth factor β (TGFβ) family and is produced by osteoprogenitor cells, osteoblasts, and chondroblasts. Among the various subtypes, BMP2, BMP4, BMP5, and BMP6 have been shown to be overexpressed in tumor and stromal cells, as demonstrated by immunohistochemical studies [17,19,20]. Noh et al. reported the overexpression of BMP9, osteocalcin, and osteopontin in both tumor and stromal cells [16]. Nagano et al. also noted the expression of BMP2, osteocalcin, and osteonectin by metastatic tumor cells in lymph nodes [14]. These bone-producing factors transform mesenchymal cells into osteoblasts, leading to heterotopic bone formation. These suggestions are further supported by the findings of Ann-Marie et al., who observed a heterogeneous staining pattern for BMP2, with expression in tumor and mesenchymal cells primarily surrounding the bone [17].

In addition, KRAS mutations in colorectal adenocarcinoma with OM were detected in three cases by Liu et al. and one case by Imaeda et al. [5,21] However, the association between OM and KRAS mutations is not established.

OM poses a diagnostic challenge since it can mimic mesenchymal tumors like osteosarcoma and the metastasis of carcinoma to the bone. Therefore, meticulous examination of histopathologic sections is crucial, as OM shows benign features but malignant stromal cells are seen in osteosarcoma [21]. Image correlation would be helpful in differentiating between OM and metastasis to the bone. In the present case, the metaplastic bone consists of benign osteocytes and is confined to the muscle proper of the colon wall, ruling out metastasis to the bone. There are no adequate studies regarding prognostic significance OM in colonic adenocarcinoma for individual patients therefore, it requires further study.

## 4 Conclusion

To our knowledge, this is the first report of OM in colon adenocarcinoma in an Ethiopian patient. OM should be differentiated from mesenchymal tumors like osteosarcoma and metastasis of carcinoma to the bone, as the latter two have worse prognoses. Further studies are necessary to determine the etiology, pathogenesis, and prognostic significance of OM in colon adenocarcinoma.

## Abbreviations

BMP: bone morphogenetic protein
DM: diabetes mellitus
GIT: gastrointestinal tract
OM: osseous metaplasia
RLQ: right lower quadrant
TGFβ: transforming growth factor β

## Patient consent statement

Written informed consent was obtained from the patient's next of kin for publication of this case report and accompanying images.

## Ethics approval

The study was notified to the university ethics committee; but this is a case report and it does not need a specific ethical approval.

## Guarantor

Seblewengel Maru Wubalem.

## Funding

This work did not receive any specific grant from funding agencies in the public, commercial, or not-for-profit sectors.

## Author contribution

1. Seblewengel Maru Wubalem: Conceptualization; Data curation; Resources; Visualization; writing – original draft; Writing – review and editing
2. Birhanu Kassie Reta: Data curation; Visualization; Writing – review and editing
3. Woldie Jember Zewdie: Visualization; Writing – review and editing
4. Shemsu Abraham Hussien: Data curation; Supervision; Visualization; Writing – original draft; Writing – review and editing

## Declaration of competing interest

The authors declare that they have no known competing financial interests or personal relationships that could have appeared to influence the work reported in this paper.
